# DeCban: Prediction of circRNA-RBP Interaction Sites by Using Double Embeddings and Cross-Branch Attention Networks

**DOI:** 10.3389/fgene.2020.632861

**Published:** 2021-01-22

**Authors:** Liangliang Yuan, Yang Yang

**Affiliations:** ^1^Department of Computer Science and Engineering, Center for Brain-Like Computing and Machine Intelligence, Shanghai Jiao Tong University, Shanghai, China; ^2^Key Laboratory of Shanghai Education Commission for Intelligent Interaction and Cognitive Engineering, Shanghai, China

**Keywords:** circular RNAs, RNA binding proteins, deep learning, double embeddings, attention network

## Abstract

Circular RNAs (circRNAs), as a rising star in the RNA world, play important roles in various biological processes. Understanding the interactions between circRNAs and RNA binding proteins (RBPs) can help reveal the functions of circRNAs. For the past decade, the emergence of high-throughput experimental data, like CLIP-Seq, has made the computational identification of RNA-protein interactions (RPIs) possible based on machine learning methods. However, as the underlying mechanisms of RPIs have not been fully understood yet and the information sources of circRNAs are limited, the computational tools for predicting circRNA-RBP interactions have been very few. In this study, we propose a deep learning method to identify circRNA-RBP interactions, called DeCban, which is featured by hybrid double embeddings for representing RNA sequences and a cross-branch attention neural network for classification. To capture more information from RNA sequences, the double embeddings include pre-trained embedding vectors for both RNA segments and their converted amino acids. Meanwhile, the cross-branch attention network aims to address the learning of very long sequences by integrating features of different scales and focusing on important information. The experimental results on 37 benchmark datasets show that both double embeddings and the cross-branch attention model contribute to the improvement of performance. DeCban outperforms the mainstream deep learning-based methods on not only prediction accuracy but also computational efficiency. The data sets and source code of this study are freely available at: https://github.com/AaronYll/DECban.

## 1. Introduction

Circular RNAs (circRNAs) are a special kind of non-coding RNA molecules. Different from linear RNAs, circRNA molecules have closed-ring structures, which are not affected by RNA exonuclease, and their expression is more stable (Pamudurti et al., 2017; Li et al., 2018). Although natural circRNAs were discovered more than two decades ago, their important roles in gene regulation and disease development have just been revealed in recent years (Hansen et al., 2013; Li et al., 2015).

Emerging studies have shown that circRNAs can bind to various types of proteins to affect protein localization, regulate protein expression, or influence protein-protein-interactions. The circRNA-binding-proteins (circRBPs) include transcription factors, RNA processing proteins, proteases, and common RNA-binding-proteins (RBPs) that can be bound with linear RNAs. Understanding the interactions between circRNAs and proteins is helpful for revealing the biological functions of circRNAs (Du et al., 2017; Zang et al., 2020). For the past decade, high-throughput experimental technologies have been widely used to detect the interactions between RNAs and proteins, like cross-linking and immunoprecipitation followed by RNA sequencing (CLIP-Seq) (Yang et al., 2015). The large-scale experimental data makes it possible to predict RNA-protein interactions (RPIs) based on machine learning methods (Li et al., 2013). Compared with expensive and time-consuming wet experiments, the computational methods have considerably sped up the identification of interactions, thus the automatic prediction of RPI has been a hot topic in the bioinformatics field (Pan et al., 2019).

The existing prediction tools include both RNA-oriented or protein-oriented, i.e., identifying the binding sites in the RNA chain and protein chain, respectively (Yan et al., 2016). Benefitting from the abundant domain knowledge from protein databases, many studies perform prediction based on protein information. By contrast, much fewer studies focus on the binding sites on circRNAs (Ju et al., 2019; Zhang et al., 2019; Jia et al., 2020; Wang and Lei, 2020). The reasons are two-folds. For one thing, compared with other non-coding RNAs, like microRNAs and long non-coding RNAs, research on circRNAs has been largely lagged and their data is scarce. For another thing, the prediction for circRNAs is a very difficult task, due to the long sequences, sparsely distributed binding sites and limited information that could be extracted.

As circRNAs have attracted more and more attention, experimental data of circRNAs has increased rapidly. Till now, a lot of circRNA-protein interactions have been revealed and released in public databases, e.g., CircInterome that houses the RBP/miRNA-binding sites on human circRNAs (Dudekula et al., 2016). Thanks to the fast-growing circRNA data and the rise of deep learning, methods for predicting circRNA-RBP binding sites are emerging. For instance, Zhang et al. (2019) proposed a method called CRIP to predict circRNA-RBP binding sites, which is a hybrid architecture of convolutional neural networks (CNNs) and recurrent neural networks (RNNs); Jia et al. (2020) proposed an ensemble classifier, PASSION, which combines various statistical sequence features and performs feature selection to enhance the prediction accuracy.

Note that learning long sequences has still been an open problem for neural networks. Biological sequences are much longer than natural language sentences, conventional learning models, including long short-term memory networks (LSTMs) which were designed to handle long-term dependencies (Hochreiter and Schmidhuber, 1997), do not work well for extremely long sequences. Therefore, most of the existing predictors take short segments instead of full-length non-coding RNAs as input to identify the binding sites (Pan and Shen, 2017, 2018; Pan et al., 2018; Zhang et al., 2019), i.e., they divid the RNA sequences into short fragments and predict whether a fragment is a binding site or not. Obviously, such simplification does not accord with the real scenario. For one thing, RPIs are usually determined by the full-length RNA information rather than short fragments; and for the other thing, the binding regions only make up a tiny proportion in the whole RNA sequences, while the fragment-based prediction often constructs relatively balanced datasets, leading to a high false-positive-rate. Therefore, to address the sparse distribution of binding sites and reduce false positive predictions, this study aims to develop a model which allows full-length circRNA sequences as input and provides reliable predictions.

Generally, the performance of machine learning methods depends on two factors, namely feature extraction and learning model. In traditional learning methods, RNA sequences are represented by statistical features, like the frequency of *k*-mers and secondary structure elements (Zhang et al., 2011; Chen et al., 2014). With the rise of deep learning, hand-crafted feature extraction has been largely replaced by automatic feature learning and pre-training via large-scale unlabeled datasets (Clauwaert and Waegeman, 2019; Meher et al., 2019). Word embedding is an emerging technique for representing biological sequence features. Unlike traditional features or one-hot encoding, word embedding is a kind of continuous distributed features. Commonly used word embedding methods include Word2vec (Mikolov et al., 2013), Glove (Pennington et al., 2014), ELMo (Peters et al., 2018), GPT (Radford et al., 2018), and Bert (Devlin et al., 2018). The first two models yield static embedding, i.e., the embedding vector for each word is context-independent and fixed after training (Peters et al., 2018), while the latter three methods yield context-dependent embedding vectors.

At present, static embeddings learned by shallow models have been widely used in biological sequence analysis, while only a few studies applied dynamic embedding, like Elmo and Bert. One reason is that the models based on deep learning models such as Elmo and Bert are very computation-intensive. Especially, non-coding RNA sequences are much longer than protein sequences, thus learning dynamic embedding for RNA sequences may require more complex model. In this study, we also adopt static word embedding method to represent circRNA sequences. To better mine the sequence information, we propose a double-embedding method to expand the feature space, which is further learned by deep neural networks to extract abstract features for classification.

As circRNAs are usually thousands of nucleotides, to handle the extremely long sequences, specialized model design is also required. Previous studies mainly used CNN (Alipanahi et al., 2015), RNN, or CNN-RNN hybrid models (Pan and Shen, 2017; Zhang et al., 2019). As aforementioned, these models take short fragments as input and construct balanced datasets, while true binding sites are very rare. In this study, we design a new model called DeCban (Double embedding and Cross-branch attention network) to predict the presence of RBP-binding sites on full-length circRNAs. This predictor is featured by not only a new sequence encoding scheme, i.e., double embedding, but also a cross-branch attention neural network. The network extracts sequence features of different abstract levels and different granularities, and the attention module allows the network to focus on important features for discrimination. Compared with the existing RPI prediction tools and mainstream deep learning models, DeCban has great advantages on both prediction accuracy and computational efficiency.

## 2. Methodology

### 2.1. Datasets

To evaluate the prediction performance of DeCban, we collect circRNAs and their interacting proteins from Circular RNA Interactome (https://circinteractome.nia.nih.gov/) (Dudekula et al., 2016). The sequence redundancy is removed by CD-Hit (Fu et al., 2012) with threshold 0.8, resulting into 32,216 circRNA sequences, which are bound to a total of 37 RBPs. We train a binary prediction model for each RBP and construct 37 datasets. The positive-to-negative ratio of each data set is 1:1, where the positive samples are the circRNAs binding to the RBP and negative samples are the remaining ones. The circRNAs in this set range from 100 to 30,000 nt in length, 90% of which are 500~7,000 nt. Therefore, to avoid the potential bias brought by too short and too long sequences, we only include the sequences falling in the range of 500~7,000 nt in the final data set. The data statistics are shown in Table 1.

**Table 1:** Experimental datasets.

**RBP**	**Train#**	**Test#**	**RBP**	**Train#**	**Test#**
AGO1	33547	14377	IGF2BP2	59467	25485
AGO2	57697	24724	IGF2BP3	83120	35622
AGO3	8570	3672	LIN28A	50769	21757
ALKBH5	4497	1927	LIN28B	21601	9257
AUF1	3045	1305	METTL3	9033	3871
C17ORF85	6225	2667	MOV10	6309	2703
C22ORF28	15680	6720	PTB	67963	29127
CAPRIN1	15503	6643	PUM2	4903	2101
DGCR8	57651	24707	QKI	3036	1300
EIF4A3	25017	10721	SFRS1	36563	15669
EWSR1	13253	5679	TAF15	3580	1534
FMRP	79392	34024	TDP43	2610	1118
FOX2	2756	1180	TIA1	5127	2197
FUS	60699	26013	TIAL1	9613	4119
FXR1	2908	1246	TNRC6	3876	1660
FXR2	15400	6600	U2AF65	16236	6958
HNRNPC	2588	1108	WTAP	1517	649
HUR	73352	31436	ZC3H7B	30175	12931
IGF2BP1	66355	28437			

### 2.2. Model Architecture

**Figure 1:**
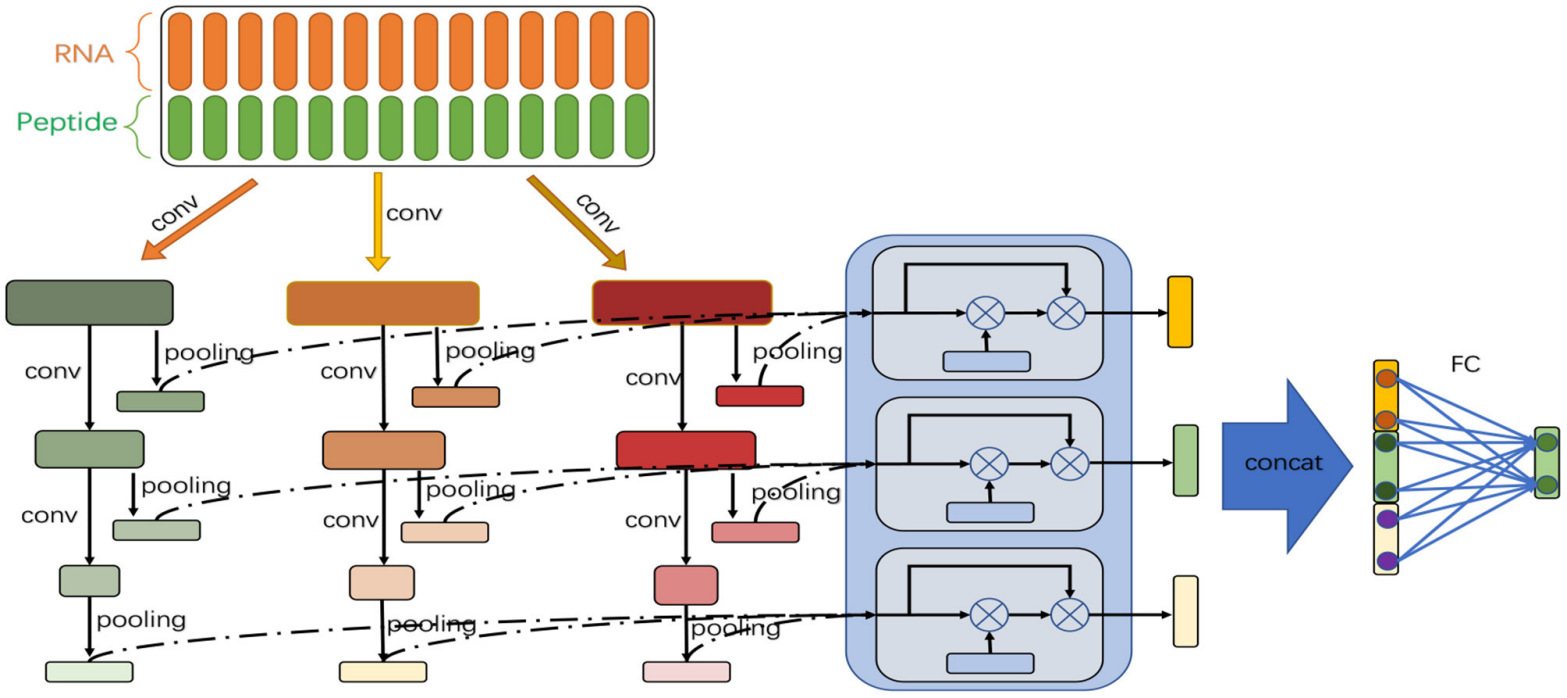
Model architecture of DeCban. The network consists of three convolutional layers and three branches (shown in green, orange, and red, respectively). An attention layer (shown in light blue) is used to integrate the outputs of the three branches. Then, the feature embeddings learned by the three layers are concatenated and fed to a fully connected layer to yield the final output.

Figure 1 shows the model architecture. The feature vectors generated by double embeddings are fed into a CNN-based neural network with multiple branches of different granularities. We introduce the self-attention mechanism to automatically integrate the semantic information extracted from different branches at each abstract level (an abstract level corresponds to a convolutional layer), and combine multiple levels of semantic information to determine whether binding sites exist in the RNA equences.

#### 2.2.1. Double Embeddings

To work with deep neural networks (DNNs), input sequences are usually converted into numerical vectors by encoding schemes, such as one-hot, which encodes each nucleotide by a four-dimensional binary vector with only one element equal to 1. One-hot is unable to express the association between different nucleotides or context information, and the low dimensionality of its feature space limits the performance of further learning by DNN. By contrast, word embeddings, that are continuous dense vectors capturing semantic association of words, have been a mainstream method to represent words and sentences in natural language processing. The training of word embeddings is based on the language modeling task, like next-word prediction, which does not require sequence labels. Thus, the training of embeddings can be performed on large-scale unlabeled corpus.

In recent years, word embeddings for *k*-mers have emerged in various bioinformatics applications. Here we also adopt word embeddings to represent circRNA sequence features. Besides, we notice that the word embedding technology has been applied more and achieved better performance in protein classification tasks, perhaps due to the bigger alphabet size and much shorter length of amino acid sequences compared with DNA/RNA sequences. To expand the alphabet, Zhang et al. (2019) developed a codon-based encoding scheme for circRNA sequences. A major advantage of this scheme lies in the enlarged feature space, as the classic one-hot has only 4 symbols while the codon-based encoding has 21 symbols, which are a combination of 3 nucleotides. The genetic codes define not only the alphabet of the new symbol system, but also the rules of correspondence between combinations of nucleotides and new symbols. Zhang et al. (2019) also showed that the three-nucleotide combinations defined by codons, are superior to random combinations defined in other encoding systems. Inspired by this idea, we convert RNA segments into pseudo-peptides and obtain word embeddings for them (we call them “pseudo-” because them are not real peptides). Then, we combine the two kinds of embeddings to generate the input features of our model. We call the new feature extraction method as double embeddings.

**Figure 2:**
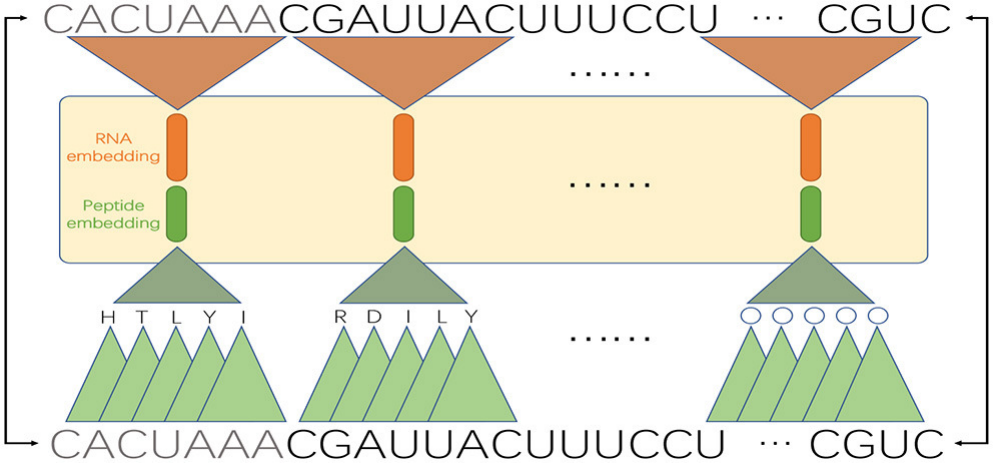
An example of double embeddings. An RNA sequence is segmented into 7-mers, and each 7-mer is converted into an embedding vector; meanwhile, the 7-mer is mapped to a pseudo-peptide, which is also converted into an embedding vector. The two embedding vectors are concatenated as a whole input.

For a circRNA fragment of length *k*, there are (*k* − 2) consecutive codons, where the codons are translated in an overlapping manner to retain more local context information. Then we perform pre-training of the word embeddings for *k*-mer RNA segments and (*k* − 2)-mer peptides, respectively. Since circRNA sequences are very long, to reduce the length of sentences, we need to set a large *k*, and long fragments also contain more local sequence information. However, training long words will require intensive computation resource. As a tradeoff, we set *k* to 7. We treat the segmented *k*-mers as words and adopt the GloVe algorithm to train their embeddings. Like NLP applications, to produce good embedding vector for words, a large corpus of text is required. Here we adopt the whole human genome as the corpus for RNA sequences (we replace “T” with “U” to convert DNAs to RNAs) and UniRef50 (https://www.uniprot.org/help/uniref) as the corpus for amino acid sequences. Finally, we construct the input matrix by using pre-trained word embeddings. Specifically, for each 7-mer fragment of a circRNA sequence, we concatenate the RNA embedding and the corresponding pseudo-peptide embedding. For example, as shown in Figure 2, the first 7-mer “CACUAUA” contains the codons CAC, ACU, CUA, UAU, and AUA, which encode the amino acids H, T, L, Y, and I, respectively. Then, the embedding vectors of “CACUAUA” and “HTLYI” are concatenated to represent the feature vector of “CACUAUA.”

Formally, for a given circRNA, let its length be *L*, which is divided into *m* segments (*m* = *L*/*k*}). Let the RNA and peptide embedding vectors for *w*_*i*_ are *R*_*i*_ and *P*_*i*_, whose dimensions are *p* and *q*, respectively. Then the double embedding for *w*_*i*_ is defined as,

(1) $$\begin{array}{l} D_{i}=R_{i}㊉P_{i},i\in \left\{1,2,\cdots \;,m\right\}, \end{array}$$

where ㊉ denotes the concatenation operation. Then the circRNA is represented by a matrix of size (*p* + *q*) × *m*, i.e., [*D*_1_, *D*_2_, ⋯ , *D*_*m*_].

#### 2.2.2. Cross-Branch Attention Network

As shown in Figure 1, the network has multiple branches, which have the same number of convolutional layers but vary in convolution kernel size. Thus, the branches can extract features at different granularities.

Besides, at the same layer of all branches, we introduce the self-attention mechanism. As the length of the input sequences varies greatly, the best features extracted from different sequences may come from different branches. The self-attention module enables the network to assign weights to the branches and obtain weighted average features. We introduce such modules in each layer to extract features of different abstract levels. Therefore, we name the model cross-branch attention network.

Formally, let the input of the network be *X*, and the first layer outputs of the three branches be $X_{1}^{1},X_{2}^{1}$, and $X_{3}^{1}$, respectively, which can be expressed as,

(2) $$\begin{array}{l} X_{j}^{1}=f\left(W_{j}^{1}*X+b_{j}^{1}\right),j\in \left\{1,2,3\right\}. \end{array}$$

Similarly, for each subsequent layer *i*, the outputs $X_{j}^{i}$ are computed as,

(3) $$\begin{array}{l} X_{j}^{i}=f\left(W_{j}^{i-1}*X_{j}^{i-1}+b_{j}^{i}\right),i\in \left\{2,3\right\},j\in \left\{1,2,3\right\}. \end{array}$$

The $X_{j}^{i}$s are further processed via a maximum pooling operation, i.e.,

(4) $$\begin{array}{l} Y_{j}^{i}=h\left(X_{j}^{i}\right), \end{array}$$

where *h*(·) is the max-pooling function. Then, the self-attention module works on each layer to integrate the outputs of three branches,

(5) $$\begin{array}{l} Y_{attn}^{i}\left(W_{a},Y_{1:3}^{i}\right)=SoftMax\left(g\left(W_{a}*{\left(Y_{1:3}^{i}\right)}^{T}\right)\right)*Y_{1:3}^{i}, \end{array}$$

where *g*(·) denotes the activation function, and $Y_{attn}^{i}$ is the output yielded by the attention module for the *i*-th layer. The outputs of the three layers are combined as *y*_*out*_,

(6) $$\begin{array}{l} Y_{out}=concat\left(Y_{attn}^{1},Y_{attn}^{2},Y_{attn}^{3}\right). \end{array}$$

Finally, the output *O* of the network is obtained through a FC layer,

(7) $$\begin{array}{l} O=SoftMax\left(g\left(W_{fc}*Y_{out}+b_{fc}\right)\right). \end{array}$$

## 3. Experimental Results

### 3.1. Experimental Settings

The DeCban model has three branches, and the sizes of their convolution kernels are 3, 5, 7, respectively. Each branch has three convolutional layers and each layer has 100 filters. The initial parameters of each attention module are randomly generated with normal distribution. We use Adam optimizer with learning rate of 0.001 to optimize the model. The number of early stopping rounds is set to 10, and the training-to-test ratio is 7:3.

### 3.2. Baseline Methods

To assess the performance of DeCban, we compare it with not only the existing predictor for RNA-protein interactions but also mainstream deep neural networks, including a recent method called CRIP (Zhang et al., 2019), recurrent neural networks (RNNs), and convolutional neural networks (CNNs). Note that CRIP performs prediction on short fragments (i.e., 101-nt), thus for a full-length RNA sequence, we first divide it into fragments and use CRIP to predict for each fragment, and then merge the results to get the prediction for the whole sequence. The other baseline models fall into five groups. Each group contains three methods with the same backbone model but different feature representations, namely RNA embeddings, peptide embeddings, and double embeddings. In addition, the performance of DeCban working with RNA or peptide embeddings alone is evaluated. The specification of baseline models is as follows.

- Group 1—LSTM: a vanilla long short-term memory network (Hochreiter and Schmidhuber, 1997).
- Group 2—BiLSTM with attention: a bidirectional LSTM with attention mechanism (Zhou et al., 2016)^1^.
- Group 3—TextCNN: a TextCNN (Kim, 2014) model.
- Group 4—ResNet18 base: a basic ResNet18 model (He et al., 2016).
- Group 5—ResNet18 small: a simplified ResNet18 model, which has the same architecture as ResNet18 but fewer convolutional kernels on each layer.
- CRIP: a CNN-RNN hybrid model for the prediction of RBP-bindings sites on RNAs (Zhang et al., 2019).

### 3.3. Experimental Results and Analysis

For a comprehensive comparison, we consider not only the prediction accuracy but also computational efficiency. The accuracy is evaluated by the common metrics of machine learning models, F_1_ and AUC score (Area under the ROC Curve). The efficiency is assessed by the number of parameters and speedup.

**Figure 3:**
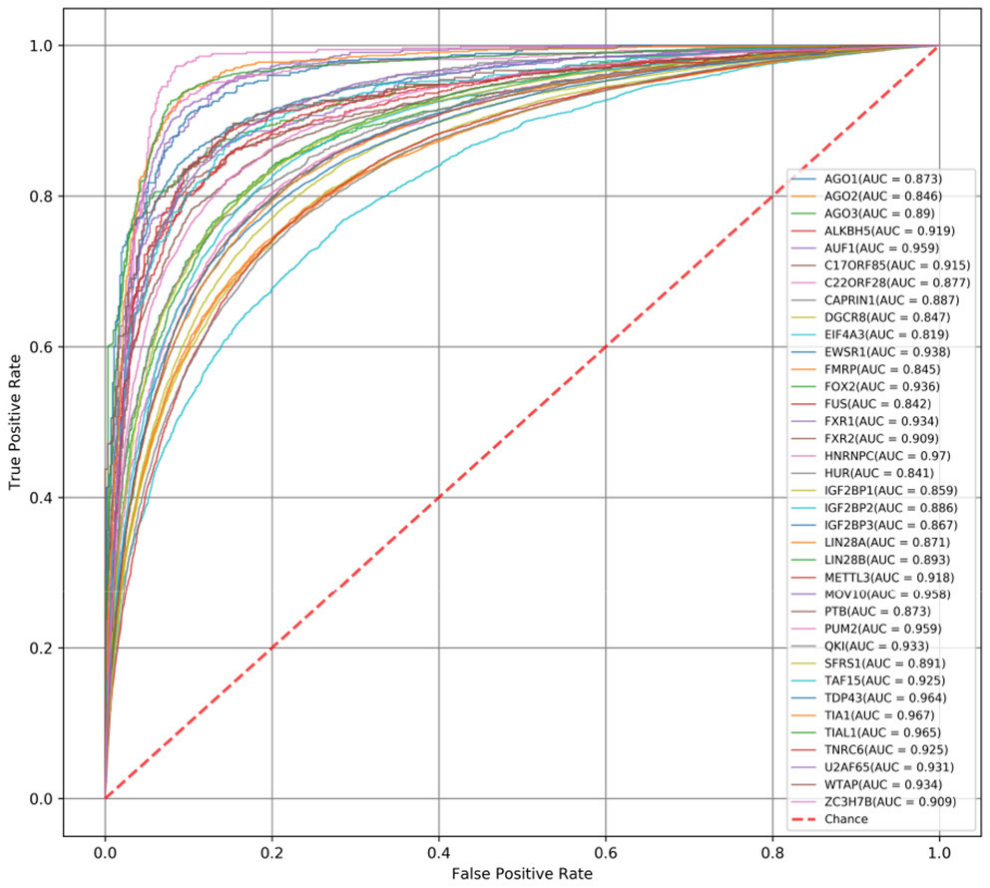
The ROC curves obtained by DeCban for 37 circRNA data sets.

**Figure 4:**
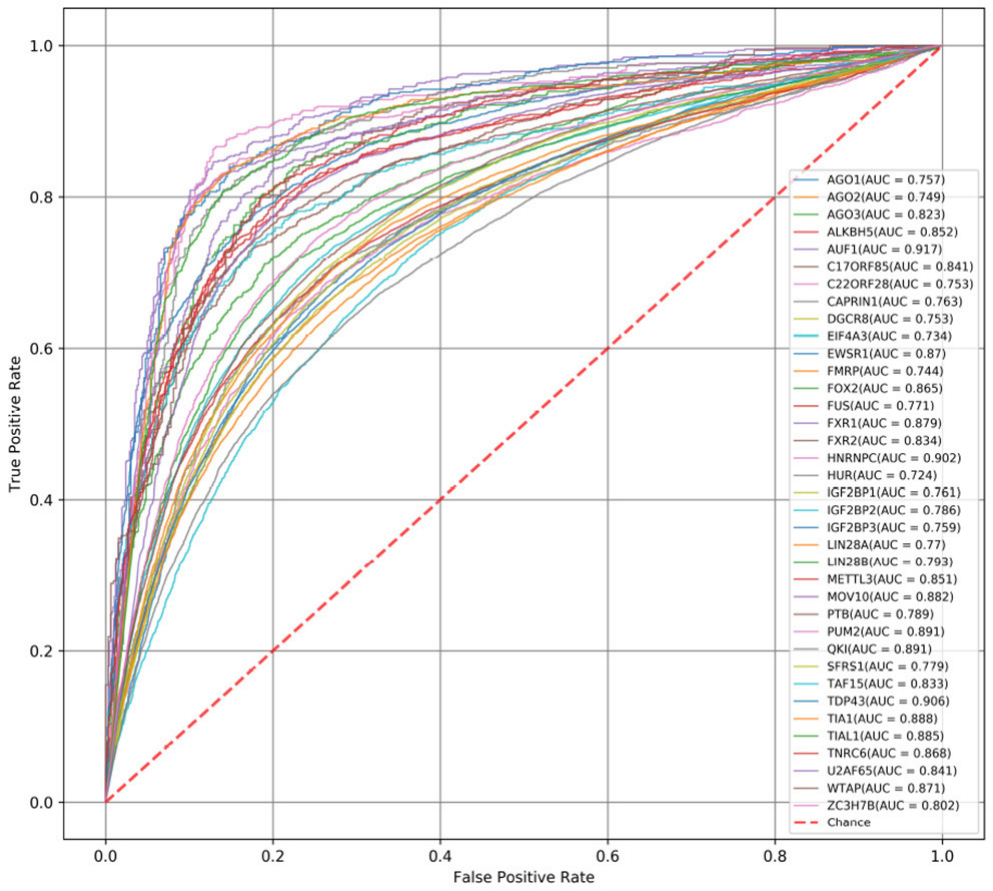
The ROC curves obtained by CRIP for 37 circRNA data sets.

First, we compare the AUC scores of DeCban and CRIP on all 37 data sets. The ROC curves are shown in Figures 3, 4, respectively. The AUC scores range from 0.819 to 0.970, and the average AUC is 0.905. The lowest, highest, and average AUCs of these two methods are 0.819 vs. 0.734, 0.970 vs. 0.917, and 0.905 vs. 0.821, respectively. DeCban has an obvious advantage over CRIP.

Second, we compare the F_1_ scores for all baseline models. Table 2 shows the average F_1_, number of parameters and speedup. As can be seen, DeCban achieves the highest average F_1_ of 0.841, and the second best model is BiLSTM with attention, whose average F_1_ is 0.827. The detailed scores for all 37 data sets are listed in Supplementary Table 1. DeCban obtains the highest F_1_ scores on all of the datasets. Meanwhile, DeCban has a lightweight architecture. Compared with the second best model BiLSTM, DeCban has a significant reduction on model parameters. The detailed comparison results are discussed in sections 3.3.1–3.3.5.

**Table 2:** Experimental results of different models^a^.

**Model**		**Param^**b**^**	**Avg F^**a**^**	**Speedup^**c**^**
LSTM-base	RNA	118 K	0.685	1.8x
	Peptide	132 K	0.685	2.0x
	Double	183 K	0.692	3.3x
BiLSTM-attention	RNA	647 K	0.817	6.4x
	Peptide	676 K	0.810	6.4x
	Double	778 K	0.827	8.2x
CNN-base	RNA	26 K	0.796	1.0x
	Peptide	30 K	0.793	1.2x
	Double	46 K	0.806	2.3x
ResNet-18-base	RNA	3,914 K	0.811	2.7x
	Peptide	3,927 K	0.803	2.6x
	Double	3,972 K	0.814	3.7x
ResNet-18-small	RNA	254 K	0.770	1.7x
	Peptide	255 K	0.761	1.8x
	Double	261 K	0.773	2.7x
CRIP	–	900 K	0.766	5.7x
DeCban	One-hot	33 K	0.822	9.6x
	RNA	79 K	0.833	1.8x
	Peptide	93 K	0.826	2.0x
	Double	141 K	0.841	3.2x

a RNA, Peptide, Double denote the RNA embedding, Peptide embedding, and double embedding, respectively.

b Param denotes the number of parameters in the model.

c *Speedup measures the relative performance of two methods processing the same problem in terms of speed. We use CNN-base with RNA embedding as the basic reference, i.e., its speedup is 1.0x*.

#### 3.3.1. Comparison of the Sequence Encoding Methods

From Table 2, it can be observed that double embeddings can improve the performance of both baseline models and DeCban. Compared to original RNA embeddings, double embeddings increase F_1_ by around 1%. In the meantime, using double embeddings do not significantly increase the complexity of the model. The total number of parameters of DeCban using double embeddings has the same order of magnitude as that of the model with RNA embeddings or amino acid embeddings. Taking ResNet-18-base as an example, the number of parameters is increased by <1.5%, while the average F_1_ on 37 data sets is increased by nearly 1 percentage.

The results suggest that the combination of RNA information and pseudo-peptide information can improve the data representation ability, although the “peptides” are not biological meaningful. A major reason for the performance improvement is the enlarged feature space. Moreover, the new encoding method traverses the RNA *k*-mers sequentially in an overlapping manner, thus retaining some local context information, which may be helpful for capturing the dependency relationship of nucleotides.

In addition, we replace the double embedding encoding with the traditional one-hot encoding for comparison. The average *F*_1_ on 37 data sets is 0.822, and the training speed is significantly slower than double embedding. This result shows the advantages of double embedding over traditional one-hot encoding.

#### 3.3.2. Comparison of Model Architectures

As DeCban is a convolutional neural network, we compare it with the state-of-the-art CNN model, ResNet-18. The number of layers and parameters of ResNet is much larger than that of DeCban. Specifically, the parameter amount of ResNet-18-base is 28 times of DeCban, while the F_1_ score is 2.5% lower than DeCban. Considering that ResNet might overfit the data due to the large model size, we implement a lightweight version of ResNet-18, namely the ResNet-18-small, by reducing the number of convolutional kernels for each layer, then the amount of parameters is at the same order of magnitude as DeCban. However, after the simplification, the prediction accuracy drops significantly. Comparing with ResNet-18-base, the F_1_ scores of three embedding methods are decreased by 0.038, 0.043, and 0.044, respectively. By contrast, benefitting from the multi-branch and self-attention mechanism, DeCban can extract features of different scales, and achieve better accuracy with much higher efficiency. Even using only RNA word embeddings, DeCban outperforms all baseline models, demonstrating the superiority of the new model architecture.

Besides CNN models, we also consider the widely-used RNN model, LSTM. Although LSTM was designed to address the gradient vanishing issue and long-term dependencies, it is still difficult for LSTM to handle very long sequences. It can be seen from the experimental results that the performance of vanilla LSTM is poor. When using the double embeddings, the average F_1_ on 37 datasets is 0.692, which is much lower than that of basic CNN (0.806). The gap of performance between these two kinds of models may be attributed to the large difference in the sequence length.

As the input sequences vary greatly in length, a large number of meaningless zeros are filled at the end of short sequences. The padding operation affects the training of LSTM, while CNN has more flexibility in extracting features from sequences with varying length. In this case, attention mechanism becomes a necessary part to enable the model focus on informative regions, thus the BiLSTM model with attention improves the performance of LSTM significantly, even better than basic CNNs and ResNets.

As for the training speed, RNN models generally need longer training time compared with CNN-based models. BiLSTM-attention becomes the most time-consuming model. By contrast, although ResNet-18 has the most parameters, it takes only less than half of the training time of BiLSTM-attention. Thus, the CNN-based DeCban model also achieves high efficiency. Taking the DeCban using double embedding as an example, the parameters are only one fifth of those of BiLSTM-attention, but the average F_1_ value is increased by 1.4%, which shows that the proposed network can achieve better performance with less computing resources.

### 3.4. Comparison With the Latest Models

In addition to the baseline models with common model architectures, we compare DeCban with the existing predictors for RBP-RNA interactions. Currently, the predictors for circRNAs are very few. CRIP (Zhang et al., 2019) and PASSION (Jia et al., 2020) are two recently developed models. We compare them with DeCban in terms of feature extraction, model architecture, and input, as described in the following.

CRIP also uses the 3-nucleotide codons to convert RNAs into pseudo-amino acids, i.e., the stacked-codon encoding scheme. However, CRIP presents the pseudo-amino acids as one-hot vectors, while DeCban uses word embeddings for both original RNAs and the converted pseudo-amino acids. PASSION incorporates some traditional statistical features in addition to CRIP's features. Therefore, a major difference between DeCban and the previous studies is using continuous dense feature encoding instead of sparse discrete features. Besides, the double embeddings contain the information of both RNA segments and pseudo-peptides, so as to strengthen the representation of raw sequences.

As for the model architecture, CRIP adopts a CNN-LSTM hybrid network, and PASSION proposes an ensemble classifier, which combines the hybrid network with an artificial neural network (consisting of fully-connected layers). DeCban is a CNN-based multi-branch attention network. As shown in Table 2, the parameter quantity of CRIP is 900 K, and PASSION has more parameters due to the ensemble nature; while DeCban with double embedding uses only one seventh of the parameters of CRIP.

Finally, both CRIP and PASSION perform prediction on short fragments, i.e., 101-nt segments. The incomplete sequences may lose some characteristics of original RNA molecules and lead to more false positive predictions, as mentioned in Zhang et al. (2019), while DeCban handles full-length sequences. Figure 4 shows the ROC curve of CRIP. The average AUC value of the CRIP model on 37 data sets is 0.821, while DeCban is 0.905. DeCban gets significantly higher AUC value than that of CRIP on nearly all datasets. And, according to the results reported in Jia et al. (2020), PASSION's AUC is about 0.01 higher than that of CRIP. As both these two methods' inputs are short fragments with balanced positive-to-negative ratio, they may have close performance when handling full-length circRNAs.

## 4. Discussion

Circular RNAs are a special kind of non-coding RNAs, which play an important role in gene regulation and disease development. Studying the interactions between circRNAs and RBPs can reveal the functions of circRNAs. However, the prediction of binding sites on circRNAs faces many challenges.

First, the length range of circRNA sequences is very large, from tens to over 100,000 nt, which adds great difficulty to the learning models. Thus, it is important to design a network to adapt to the large variance of input sequences. The multi-branch design of DeCban aims to extract features from different ranges of sequence regions, as the branches differ in kernel sizes, leading to different receptive fields. For instance, assume that step length is 3, with 0 padding and 0 dilation. When the convolution kernel size is 3, the receptive field sizes of the features output by the first and second layers are 3 and 5, respectively. When the convolution kernel size is 5, the receptive field sizes of the features output by the first and second layers are 5 and 9. Thus, different convolution kernel sizes can extract features of different scales.

The second challenge is that RBP-binding sites are extremely sparsely located in the whole RNA sequences, i.e., the number of binding sites are few and the binding regions are very short compared to full-length sequences. Thus, this is a severely imbalanced learning task, as most of the regions have no binding affinity. The attention mechanism in DeCban can alleviate this problem to a certain extent, which enables the model focus on key regions in long sequences.

The third challenge arises from the data side. Compared with linear RNAs, domain knowledge or information sources other than sequences are lacked. By utilizing the codon-based mapping between RNA and peptides, and performing large-scale pre-training of word embeddings for both RNA segments and peptides, we propose a new feature representation method for circRNAs, called double embeddings. Experiments show that this method effectively improves the representation ability for raw sequences.

Compared with the existing circRNA-RBP prediction methods, DeCban has the following advantages:

1. The prediction can be performed on full-length circRNA sequences instead of short segments.
2. The model is highly efficient, whose training has a low cost on computation resources.
3. The high prediction accuracy makes it a useful tool for studying circRNA-RBP interactions.

## 5. Conclusion

In this study, we propose a method called DeCban to predict the binding relationship between RNA-binding-proteins and circRNAs. Different from the existing tools which can only handle short segments of circRNAs, DeCban is able to predict whether a binding site is present on full-length circRNAs. In order to solve the problem of large length span and sparse distribution of binding sites, we design a multi-branch and multi-layer convolutional neural network with an attention module. Moreover, to enhance the input data representation, we propose the double embedding encoding scheme, which is superior to the traditional single RNA embedding due to the introduction of amino-acid-level sequence information. We perform experiments on 37 data sets, corresponding to 37 RBPs. The experimental results show that our method achieves the best results compared with a variety of advanced deep learning structures. DeCban will be a useful tool for studying the interactions between RBP and circRNA.

## Data Availability Statement

The original contributions presented in the study are included in the article/Supplementary Materials, further inquiries can be directed to the corresponding author/s.

## Author Contributions

LY and YY designed the model, analyzed the results, and wrote the manuscript. LY conducted the experiments. All authors contributed to the article and approved the submitted version.

## Conflict of Interest

The authors declare that the research was conducted in the absence of any commercial or financial relationships that could be construed as a potential conflict of interest.

## Footnotes

^1^An advanced model structure based on LSTM, which has achieved state of art results on multiple NLP tasks.

## References

[B1] Alipanahi B. Delong A. Weirauch M. T. Frey B. J. (2015). Predicting the sequence specificities of DNA- and RNA-binding proteins by deep learning. Nat. Biotechnol. 33:831. 10.1038/nbt.3300 26213851

[B2] Chen W. Lei T. Y. Jin D. C. Lin H. Chou K. C. (2014). Pseknc: a flexible web server for generating pseudo k-tuple nucleotide composition. Anal. Biochem. 456:53. 10.1016/j.ab.2014.04.001 24732113

[B3] Clauwaert J. Waegeman W. (2019). Novel transformer networks for improved sequence labeling in genomics. bioRxiv [Preprint]. 836163. 10.1101/836163 33125335

[B4] Devlin J. Chang M. W. Lee K. Toutanova K. (2018). Bert: Pre-training of deep bidirectional transformers for language understanding. arXiv preprint arXiv:1810.04805.

[B5] Du W. W. Zhang C. Yang W. Yong T. Awan F. M. Yang B. B. (2017). Identifying and characterizing circRNA-protein interaction. Theranostics 7:4183. 10.7150/thno.21299 29158818 PMC5695005

[B6] Dudekula D. B. Panda A. C. Grammatikakis I. De S. Abdelmohsen K. Gorospe M. (2016). Circinteractome: a web tool for exploring circular RNAs and their interacting proteins and microRNAs. RNA Biol. 13, 34–42. 10.1080/15476286.2015.1128065 26669964 PMC4829301

[B7] Fu L. Niu B. Zhu Z. Wu S. Li W. (2012). CD-hit. Bioinformatics 28, 3150–3152. 10.1093/bioinformatics/bts565 23060610 PMC3516142

[B8] Hansen T. B. Kjems J. Damgaard C. K. (2013). Circular RNA and MIR-7 in cancer. Cancer Res. 73, 5609–5612. 10.1158/0008-5472.CAN-13-1568 24014594

[B9] He K. Zhang X. Ren S. Sun J. (2016). Deep residual learning for image recognition, in The IEEE Conference on Computer Vision and Pattern Recognition (CVPR), Doha. 10.1109/CVPR.2016.90

[B10] Hochreiter S. Schmidhuber J. (1997). Long short-term memory. Neural Comput. 9, 1735–1780. 10.1162/neco.1997.9.8.1735 9377276

[B11] Jia C. Bi Y. Chen J. Leier A. Li F. Song J. (2020). PASSION: an ensemble neural network approach for identifying the binding sites of RBPs on circRNAs. Bioinformatics 36, 4276–4282. 10.1093/bioinformatics/btaa522 32426818

[B12] Ju Y. Yuan L. Yang Y. Zhao H. (2019). Circslnn: Identifying rbp-binding sites on circrnas via sequence labeling neural networks. Front. Genet. 10:1184. 10.3389/fgene.2019.01184 31824574 PMC6886371

[B13] Kim Y. (2014). Convolutional neural networks for sentence classification. arXiv preprint arXiv:1408.5882. 10.3115/v1/D14-118131070725

[B14] Li J.-H. Liu S. Zhou H. Qu L.-H. Yang J.-H. (2013). starbase v2. 0: decoding miRNA-ceRNA, miRNA-ncRNA and protein-RNA interaction networks from large-scale clip-seq data. Nucl. Acids Res. 42, D92–D97. 10.1093/nar/gkt1248 24297251 PMC3964941

[B15] Li X. Yang L. Chen L.-L. (2018). The biogenesis, functions, and challenges of circular RNAs. Mol. Cell 71, 428–442. 10.1016/j.molcel.2018.06.034 30057200

[B16] Li Y. Zheng Q. Bao C. Li S. Guo W. Zhao J.. (2015). Circular RNA is enriched and stable in exosomes: a promising biomarker for cancer diagnosis. Cell Res. 25:981. 10.1038/cr.2015.82 26138677 PMC4528056

[B17] Meher P. K. Sahu T. K. Gahoi S. Satpathy S. Rao A. R. (2019). Evaluating the performance of sequence encoding schemes and machine learning methods for splice sites recognition. Gene 705, 113–126. 10.1016/j.gene.2019.04.047 31009682

[B18] Mikolov T. Chen K. Corrado G. Dean J. (2013). Efficient estimation of word representations in vector space. Comput. Sci. arXiv preprint arXiv:1301.3781.

[B19] Pamudurti N. R. Bartok O. Jens M. Ashwalfluss R. Stottmeister C. Ruhe L.. (2017). Translation of circrnas. Mol. Cell 66, 9–21.e7. 10.1016/j.molcel.2017.02.021 28344080 PMC5387669

[B20] Pan X. Rijnbeek P. Yan J. Shen H.-B. (2018). Prediction of rna-protein sequence and structure binding preferences using deep convolutional and recurrent neural networks. BMC Genomics 17:582. 10.1186/s12864-018-4889-1 29970003 PMC6029131

[B21] Pan X. Shen H.-B. (2017). RNA-protein binding motifs mining with a new hybrid deep learning based cross-domain knowledge integration approach. BMC Bioinformatics 18:136. 10.1186/s12859-017-1561-8 28245811 PMC5331642

[B22] Pan X. Shen H.-B. (2018). Predicting RNA-protein binding sites and motifs through combining local and global deep convolutional neural networks. Bioinformatics 34, 3427–3436. 10.1093/bioinformatics/bty364 29722865

[B23] Pan X. Yang Y. Xia C.-Q. Mirza A. H. Shen H.-B. (2019). Recent methodology progress of deep learning for RNA-protein interaction prediction. Wiley Interdisc. Rev. 10:e1544. 10.1002/wrna.1544 31067608

[B24] Pennington J. Socher R. Manning C. (2014). Glove: Global vectors for word representation, in Proceedings of EMNLP, Doha, 1532–1543. 10.3115/v1/D14-1162

[B25] Peters M. E. Neumann M. Iyyer M. Gardner M. Clark C. Lee K. (2018). Deep contextualized word representations. arXiv preprint arXiv:1802.05365. 10.18653/v1/N18-1202

[B26] Radford A. Narasimhan K. Salimans T. Sutskever I. (2018). Improving language understanding by generative pre-training.

[B27] Wang Z. Lei X. (2020). Matrix factorization with neural network for predicting circrna-rbp interactions. BMC Bioinformatics 21:229. 10.1186/s12859-020-3514-x 32503474 PMC7275382

[B28] Yan J. Friedrich S. Kurgan L. (2016). A comprehensive comparative review of sequence-based predictors of DNA-and RNA-binding residues. Brief. Bioinformatics 17, 88–105. 10.1093/bib/bbv023 25935161

[B29] Yang Y.-C. T. Di C. Hu B. Zhou M. Liu Y. Song N.. (2015). CLIPdb: a CLIP-seq database for protein-RNA interactions. BMC Genomics 16:51. 10.1186/s12864-015-1273-2 25652745 PMC4326514

[B30] Zang J. Lu D. Xu A. (2020). The interaction of circRNAs and RNA binding proteins: an important part of circRNA maintenance and function. J. Neurosci. Res. 98, 87–97. 10.1002/jnr.24356 30575990

[B31] Zhang K. Pan X. Yang Y. Shen H.-B. (2019). Crip: predicting circRNA-RBP interaction sites using a codon-based encoding and hybrid deep neural networks. RNA 25:rna.070565.119. 10.1261/rna.070565.119 31537716 PMC6859861

[B32] Zhang Y. Wang X. Kang L. (2011). A k-mer scheme to predict pirnas and characterize locust piRNAs. Bioinformatics 27:771. 10.1093/bioinformatics/btr016 21224287 PMC3051322

[B33] Zhou P. Shi W. Tian J. Qi Z. Li B. Hao H. (2016). Attention-based bidirectional long short-term memory networks for relation classification, in Proceedings of the 54th Annual Meeting of the Association for Computational Linguistics, Doha. 10.18653/v1/P16-2034

